# Development of Continuous Assessment of Muscle Quality and Frailty in Older Patients Using Multiparametric Combinations of Ultrasound and Blood Biomarkers: Protocol for the ECOFRAIL Study

**DOI:** 10.2196/50325

**Published:** 2024-02-23

**Authors:** Naiara Virto, Xabier Río, Garazi Angulo-Garay, Rafael García Molina, Almudena Avendaño Céspedes, Elisa Belen Cortés Zamora, Elena Gómez Jiménez, Ruben Alcantud Córcoles, Leocadio Rodriguez Mañas, Alba Costa-Grille, Ander Matheu, Diego Marcos-Pérez, Uxue Lazcano, Itziar Vergara, Laura Arjona, Morelva Saeteros, Diego Lopez-de-Ipiña, Aitor Coca, Pedro Abizanda Soler, Sergio J Sanabria

**Affiliations:** 1 Department of Physical Activity and Sport Science Faculty of Education and Sport University of Deusto Bilbao Spain; 2 Department of Geriatrics Complejo Hospitalario Universitario de Albacete Albacete Spain; 3 Center for Biomedical Research Network on Fragility and Healthy Aging (CIBERfes) Instituto de Salud Carlos III Madrid Spain; 4 Facultad de Enfermería de Albacete Universidad de Castilla-La Mancha Albacete Spain; 5 Geriatrics Department University Hospital of Getafe Getafe Spain; 6 Biodonostia Health Research Institute Donostia Spain; 7 IKERBASQUE Basque Foundation for Science Bilbao Spain; 8 Osakidetza Health Care Department Research Unit APOSIs Gipuzkoa Spain; 9 Research Network in Chronicity Primary Care and Health Promotion (RICAPPS) Barakaldo Spain; 10 Deusto Institute of Technology University of Deusto Bilbao Spain; 11 Department of Physical Activity and Sports Sciences Faculty of Health Sciences Euneiz University Vitoria-Gasteiz Spain; 12 Facultad de Medicina de Albacete Universidad de Castilla-La Mancha Albacete Spain; 13 Department of Radiology Stanford University Palo Alto, CA United States

**Keywords:** muscle, ultrasound, blood-based biomarkers, sarcopenia, frailty, older adults

## Abstract

**Background:**

Frailty resulting from the loss of muscle quality can potentially be delayed through early detection and physical exercise interventions. There is a demand for cost-effective tools for the objective evaluation of muscle quality, in both cross-sectional and longitudinal assessments. Literature suggests that quantitative analysis of ultrasound data captures morphometric, compositional, and microstructural muscle properties, while biological assays derived from blood samples are associated with functional information.

**Objective:**

This study aims to assess multiparametric combinations of ultrasound and blood-based biomarkers to offer a cross-sectional evaluation of the patient frailty phenotype and to track changes in muscle quality associated with supervised exercise programs.

**Methods:**

This prospective observational multicenter study will include patients aged 70 years and older who are capable of providing informed consent. We aim to recruit 100 patients from hospital environments and 100 from primary care facilities. Each patient will undergo at least two examinations (baseline and follow-up), totaling a minimum of 400 examinations. In hospital environments, 50 patients will be measured before/after a 16-week individualized and supervised exercise program, while another 50 patients will be followed up after the same period without intervention. Primary care patients will undergo a 1-year follow-up evaluation. The primary objective is to compare cross-sectional evaluations of physical performance, functional capacity, body composition, and derived scales of sarcopenia and frailty with biomarker combinations obtained from muscle ultrasound and blood-based assays. We will analyze ultrasound raw data obtained with a point-of-care device, along with a set of biomarkers previously associated with frailty, using quantitative real-time polymerase chain reaction and enzyme-linked immunosorbent assay. Additionally, we will examine the sensitivity of these biomarkers to detect short-term muscle quality changes and functional improvement after a supervised exercise intervention compared with usual care.

**Results:**

At the time of manuscript submission, the enrollment of volunteers is ongoing. Recruitment started on March 1, 2022, and ends on June 30, 2024.

**Conclusions:**

The outlined study protocol will integrate portable technologies, using quantitative muscle ultrasound and blood biomarkers, to facilitate an objective cross-sectional assessment of muscle quality in both hospital and primary care settings. The primary objective is to generate data that can be used to explore associations between biomarker combinations and the cross-sectional clinical assessment of frailty and sarcopenia. Additionally, the study aims to investigate musculoskeletal changes following multicomponent physical exercise programs.

**Trial Registration:**

ClinicalTrials.gov NCT05294757; https://clinicaltrials.gov/ct2/show/NCT05294757

**International Registered Report Identifier (IRRID):**

DERR1-10.2196/50325

## Introduction

Aging is associated with a gradual decline in muscle mass and function, contributing to an increased incidence and prevalence of chronic diseases [1,2]. This, in turn, often leads to situations of multimorbidity [3] and adversely affects functional autonomy [4]. The most severe manifestation of this condition is frailty, described as “a progressive decline in physiological systems that leads to decreased reserves of intrinsic capacity. This confers extreme vulnerability to stressors and increases the risk of various adverse health outcomes” [5]. Frailty is linked to dependency, hospitalization, institutionalization, falls, poor quality of life, and mortality [6-10], along with elevated health care costs [11,12]. While standardized diagnostic criteria are not universally established, the 2 most widely accepted ones [13,14] are rooted in the phenotype construct [4]. According to this construct, frailty is diagnosed when 3 or more of the following criteria are met: unintentional weight loss, self-reported exhaustion, reduced grip strength, slow gait speed, and low levels of physical activity. Moreover, the Cumulative Deficit Model—Frailty Index encompasses cognitive, functional, emotional, and nutritional status [15,16].

The frailty phenotype, as outlined by Fried et al [4], centers around muscle dysfunction [17]. Considering that weakness, slowness, and impairment of the muscular system are characteristic features of frailty, sarcopenia is likely a crucial physiopathological contributor [13,18]. Sarcopenia is a progressive skeletal muscle disease, and its prevalence tends to increase with age. Estimates suggest that sarcopenia affects between 6% and 19% of the general population aged 60 years and older, with variations depending on the applied definition [19]. Currently, the most widely used definitions come from the European Working Group on Sarcopenia in Older People 2 (EWGSOP-2) [20], the Definition and Outcomes Consortium (DOCS) [21], and the National Institute of Health Foundation (NIHF) [22]. According to the EWGSOP-2, reduced muscle strength serves as the initial criterion for probable sarcopenia, and the diagnosis is confirmed by reduced muscle mass and quality. Furthermore, when low physical performance is identified, sarcopenia is categorized as severe [20]. The DOCS supports the inclusion of both weakness (defined by low grip strength) and slowness (defined by low usual gait speed) in the definition of sarcopenia [21]. The NIHF defines sarcopenia as a loss of strength diagnosed by low grip strength, accompanied by low muscle mass [22].

In clinical care, the assessment of muscle mass and quality involves a semiquantitative evaluation using 2D images through dual-energy X-ray absorptiometry (DXA) and a body composition estimate through bioelectrical impedance analysis (BIA) [20]. It is worth noting that these techniques can be influenced by other variables, such as skeletal mass and a high BMI [23]. Radiological imaging enables comprehensive 3D mapping of muscle composition and microstructure. The proposed methods are magnetic resonance imaging (MRI) and computed tomography (CT) sequences, allowing the assessment of adipose fraction and fibrous microstructure, among other parameters [24-26]. However, because of their high cost and the potential for patient complications, these imaging methods are presently limited to research applications or as supplementary examinations for different primary indications [27].

Sarcopenia and frailty, although connected and associated with aging, are distinct conditions. Sarcopenia primarily centers around the musculoskeletal system, while frailty is a more multifactorial condition [28,29]. Various studies indicate that the prevalence of sarcopenia among older adults with frailty is higher than the prevalence of frailty among those with sarcopenia [28,30-32]. Adverse outcomes linked to muscular decline can be mitigated, delayed, or even reversed through early detection and interventions, including nutritional support and physical exercise programs [13,18,33]. Nonetheless, there exists a need for straightforward and dependable tools that facilitate the assessment of muscle quality and its implications for frailty [28,34].

Ultrasound, as a fast, noninvasive, and cost-effective imaging modality, is gaining rapid prominence for musculoskeletal examination [35,36]. Currently, clinical ultrasound images (B-mode) enable the assessment of muscle mass and morphology, encompassing measures such as muscle thickness, pennation angle, cross-sectional area, echo intensity, and fascicle length [37-39]. Despite ongoing efforts for standardization, these measurements remain highly reliant on the expertise and skills of the operator, and they do not provide definitive results for the early staging of muscle quality loss [37,40]. Ultrasound morphometric measurements of sarcopenia in older adults have demonstrated mild to moderate associations with frailty [41]. More recently, various quantitative ultrasound techniques have surfaced, involving the analysis of echogenicity, texture parameters, elastography, and acoustic wave properties. However, their translation to clinical practice is still limited [42-51]. Artificial intelligence is presenting new opportunities to objectify musculoskeletal ultrasound, with recent studies showcasing automatic muscle segmentation, fiber angle detection, and textural discrimination of muscle microstructures [52-55].

Biological biomarkers serve as valuable tools in diagnosing and stratifying patients, as well as in comprehending the underlying pathophysiology of diseases. Oxidative stress, a proinflammatory state, and immune aging play significant roles in the connection between nonspecific biomarkers and specific biological systems related to frailty and sarcopenia [56-59]. Recent studies have delved into the intricate interrelationships among various systems that underlie frailty through multi-omics approaches [60]. As an example, the FRAILOMIC initiative used blood samples to delineate a collection of biological biomarkers, encompassing both protective and risk factors. Notably, oxidative stress, vitamin D, and the cardiovascular system were found to be associated with frailty [61]. Despite these advancements, the currently available biomarkers exhibit weak individual associations with the clinical outcomes of sarcopenia and frailty. Furthermore, their ability to detect changes after physical intervention remains largely unknown.

A limited number of studies have explored the combination of ultrasound and blood-based biomarkers. In one study, changes in circulating biomarkers corresponding to a short-term resistance exercise intervention in older adults were identified. These changes were found to be significantly related to ultrasound leg cross-sectional area [62]. Associations were discovered between combined genetic and methylation scores and ultrasound-derived skeletal muscle morphometry in older women [63]. In another cross-sectional study, ultrasound characteristics of the quadriceps femoris in patients with sarcopenia were correlated with blood and urinary biomarkers [64].

In summary, there is a lack of simple and objective screening tools for diagnosing frailty and sarcopenia [28,34]. While clinical standardization of B-mode images is essential, there is also a requirement for advancements in ultrasound technology to develop quantitative indicators for assessing muscle quality [65,66]. This study is designed to assess objective methods for evaluating muscle quality using quantitative analysis techniques based on the analysis of ultrasound raw data combined with blood-based biomarkers. Additionally, the study aims to investigate the capability of these biomarkers to detect changes in muscle quality resulting from a physical exercise intervention program in older individuals with frailty.

The primary aim of this study is to assess the feasibility of combining point-of-care quantitative ultrasound parameters with blood-based assays for evaluating muscle quality and frailty in older adults. This evaluation will encompass both hospital settings and community care, with a focus on comparing the findings with traditional clinical evaluations.

## Methods

### Study Setting

**Figure 1:**
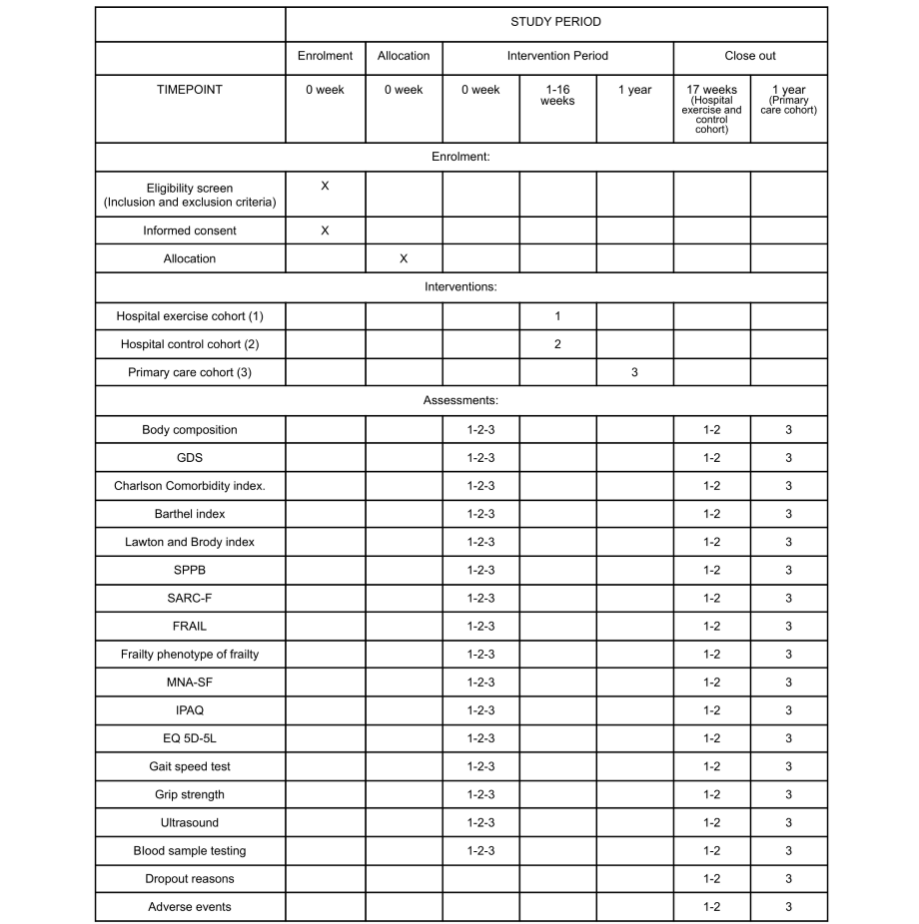
SPIRIT (Standard Protocol Items of the Recommendations for Interventional Trials) figure.

**Figure 2:**
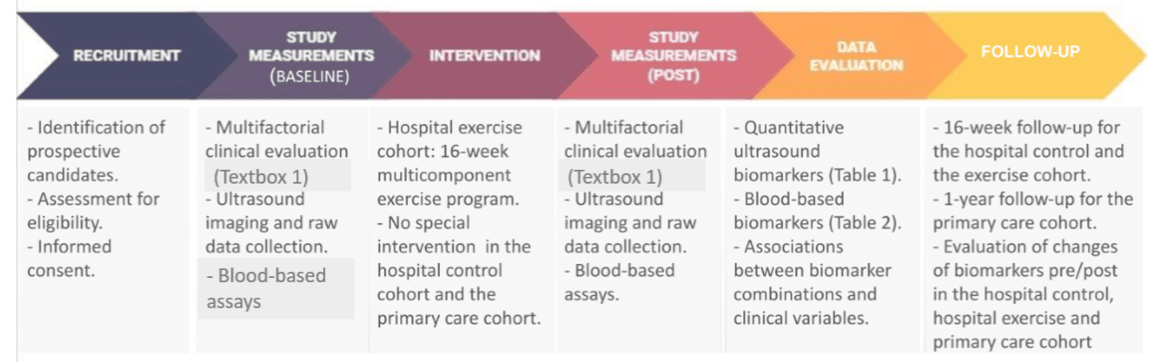
Flow diagram of the study protocol.

This study is designed as a prospective, experimental, multicenter, 3-cohort investigation. The research will be performed at Albacete University Hospital Complex, Spain (with the coordinating Clinical Research Ethics Committee; hospital 1); Getafe University Hospital, Spain (hospital 2); and primary care units of Donostialdea, Osakidetza, Spain (primary care units). The primary health care units involved in the study will be located in 2 districts/regions of Gipuzkoa: the region of Donostialdea and the region of Tolosaldea. The design of this study protocol, characterized as exploratory, adheres to the SPIRIT (Standard Protocol Items of the Recommendations for Interventional Trials; Multimedia Appendix 1) 2013 guidelines, as depicted in Figures 1 and 2.

### Study Population Recruitment

#### Hospital Exercise Cohort

Participants will be consecutively recruited from the scheduled patient list of the falls unit and the outpatient clinics of hospital 1. Upon obtaining informed consent, patients will undergo a baseline clinical evaluation. Subsequently, ultrasound measurements and a DXA scan will be conducted, and blood samples will be collected, processed, and stored. These patients will then be enrolled in a 16-week multicomponent physical exercise program (discussed later). Following the completion of the exercise program, at the 16-week follow-up, a repetition of clinical evaluation, ultrasound, DXA, and blood-sample testing will be performed.

#### Hospital Control Cohort

Participants will be consecutively recruited from the scheduled patient list of the frailty unit, day hospital, and the outpatient clinic of the Geriatrics Department of hospital 2. Upon obtaining informed consent, baseline study variables will be collected. These patients will be followed up for 16 weeks under usual care. After the follow-up period, a repetition of the baseline evaluation will be conducted.

#### Primary Care Cohort

Participants will be consecutively recruited from the scheduled patient list of the primary care units. Upon obtaining informed consent, baseline study variables will be collected. These patients will be followed up for 1 year under usual care. After the 1-year follow-up, a repetition of the baseline evaluation will be conducted.

Recruitment is conducted by the study coordinator staff at each site, under the overall supervision of the clinical principal investigator at each site. Participants will sign the informed consent at the beginning of the first visit. The professional overseeing the assessment will provide an explanation of the project, and the participant will sign both the information sheet and the informed consent to participate. The principal investigator at each site will be responsible for collecting and monitoring the documentation related to informed consent. The inclusion and exclusion criteria are listed in Textbox 1.

**Inclusion, exclusion, and termination criteria.**

**1. Inclusion criteria**

- Age of at least 70 years.
- Either gender.
- Ability to provide informed consent.
- Ability to perform all the functional tests.
- In the hospital exercise cohort, the ability to participate in the physical exercise program.

**2. Exclusion criteria**

- Expected survival of <1 year.
- Barthel Scale score <70.
- Moderate-to-severe cognitive impairment.
- Refusal to participate.
- Medical conditions that may compromise or impede follow-up assessments.
- Older adults already enrolled in regular physical exercise programs will be excluded from participating in the hospital exercise cohort.

**3. Termination criteria**

- Refusal to continue participation.
- Complications during or in between examinations and intervention.

### Allocation

The allocation sequence in this multicentric study is institution-based. Patients recruited at the Department of Geriatrics of the Complejo Hospitalario Universitario de Albacete will be automatically allocated to the supervised exercise program by default. By contrast, patients recruited at the Geriatrics Department of the University Hospital of Getafe will not be assigned to any specific interventions. Similar sociodemographic characteristics are anticipated for both patient populations. At each institution, patients will be recruited from the falls unit, outpatient clinics, and day hospital. Within each acquisition site, patient selection will be conducted based on the clinical agenda until the recruitment goals are met.

The allocation sequence is sequential, aligned with the clinical agenda, and overseen by the clinical principal investigator at each site. Any deviations from the recruitment plan outlined in the clinical agenda must be thoroughly justified and meticulously documented.

### Interventions

In the hospital exercise cohort, a 16-week supervised multicomponent physical exercise program will be implemented. The individualized exercise intervention, tailored to each individual’s functional capacity, comorbidities, and previous experience, will consist of 2 supervised sessions per week, each lasting 45 minutes. These sessions will be conducted in small groups, with 4-6 older adults per group. The sessions are divided into warm-up, main part (strength, power, and coordination exercises), and cool down (flexibility and stretching). The main part of the exercise program comprises 8 exercises targeting the major muscle groups. The progression starts with low-intensity and high-volume sessions, gradually advancing to higher intensities and lower volumes. Depending on the stage, participants will complete 2-4 sets, consisting of 4-15 repetitions, with a 30-second rest between sets and exercises. For each exercise, loads and intensities will be adjusted to achieve a total of 30 repetitions. All exercise sessions are to be conducted in the gym of the Geriatrics Department of the hospital. Participants will be encouraged to incorporate additional 90-minute walks per week into their routine. It is important to note that the exercise program is aligned with the guidelines of the VIVIFRAIL program [67], which specifically aims to prevent weakness and reduce the risk of falls.

In the hospital control and primary care cohorts, no specific intervention will be implemented.

### Retention

In alignment with the VIVIFRAIL program guidelines, our intervention program is designed to prevent weakness and reduce the risk of falls. Patients will be informed about the program’s benefits during recruitment and follow-up. Within the supervised exercise program, interventions and patient progress will be personalized and monitored, taking into account functional capacity, comorbidities, and the individual’s previous experiences. The sessions will be closely monitored at the gym of the Geriatrics Department of the hospital. The individualized sessions will enable discussions on progress with the patient throughout the intervention, fostering motivation for retention and completing the follow-up. Participants will also be encouraged to adopt healthy habits, such as incorporating an additional 90-minute walk per week. In case participants require medical care that may interfere with the ongoing study procedures, they will be managed according to the clinical routine and subsequently excluded from the study. In these cases, the data collected during the baseline examination will be used for a cross-sectional clinical evaluation of frailty and sarcopenia. However, musculoskeletal changes following the multicomponent exercise program will not be assessed.

### Safety Monitoring

Each principal investigator at every data acquisition center will oversee the monitoring and follow-up of participants included in their respective cohorts under usual care. They will make decisions regarding patient exclusion and assignment to interventions, and monitor termination criteria as deemed necessary. Additionally, participation in an exercise program is generally associated with a low risk (–1%) of adverse events [68], most of which are typically low-grade responses to exercise, such as muscle soreness [69]. However, in the event of adverse events or serious adverse events, they will be promptly reported to the Clinical Research Ethics Committees.

### Randomization and Blinding

Data analysis will be conducted independently of cohort recruitment and follow-up. The evaluation of data will take place at the Deusto Institute of Technology (Ultrasound Data Evaluation Center) and Biodonostia (Blood-based Assays Evaluation Center). Initially, only anonymized data, including ultrasound images, raw data, and blood-based assays, will be transmitted to the data evaluation centers. In the case of combination models, a training set of clinical variables will also be transferred to the data evaluation centers. Reserved data sets will be kept at the clinical acquisition centers for independent testing. This includes data from various sites and information obtained during follow-up examinations.

### Sample Size

A population of 200 participants will be recruited, with 100 participants in the primary care cohort, 50 participants in the hospital exercise cohort, and 50 participants in the hospital control cohort (2:1:1 ratio). Patient selection within each acquisition site will be conducted based on the clinical agenda until the recruitment goals are met. Each participant will undergo 2 examinations (baseline and follow-up), resulting in a minimum total of 400 examinations.

An estimated distribution of 85% of patients with frailty to 15% of healthy participants is anticipated in the hospital cohorts, while a distribution of 15% of patients with frailty to 85% of healthy participants is expected in the primary care cohort. Consequently, an accrual of 139 robust participants and 86 patients with frailty is projected. It is assumed that up to 10% of the participants may be excluded in data evaluation as a result of failed measurements or logistical challenges in collecting all variables. In cases where follow-up measurements are not feasible for patients, the baseline measurement will still be included in the evaluation of the primary outcomes. With 80% power, the planned population will enable the detection of a biomarker medium effect size of E/S=0.39, considering a 2-sided α level of .05. For the secondary goal, our sample size of 100 provides 80% power to detect a biomarker with a medium effect size (E/S=0.284) at a 2-sided α level of .05. The sample size was calculated using RiskCalc (Moody’s Analytics) [70].

### Outcomes

#### Primary Outcomes

Investigating the association between quantitative ultrasound biomarkers related to muscle mass and quality (thickness [mm], cross-sectional area [cm^2^], perimeter [mm], pennation angle [degrees]) extracted from raw data and blood-based biomarkers, as well as their combinations with clinical variables. These clinical variables encompass frailty (FRAIL [short 5-question assessment of Fatigue, Resistance, Aerobic capacity, Illnesses, and Loss of weight] scale ranging from 0 to 5, with 0 indicating robustness and 5 indicating frailty, and Frailty phenotype by Fried et al [4], ranging from 0 [robust] to 5 [frail]), sarcopenia (SARC-F [Strength, Assistance with walking, Rising from a chair, Climbing stairs, and Falls] scale scores ranging from 0 [robust] to 10 [sarcopenic]), physical function (Short Physical Performance Battery [SPPB] Scale score ranging from 0 to 12, where 0 indicates the lowest physical performance and 12 indicates the highest performance), International Physical Activity Questionnaire scores ranging from 0 (lowest) to 3 (highest), Gait Speed Test in seconds, grip strength measured by Jamar dynamometer in kilogram, disability (Global Deterioration Scale, consisting of 7 stages), Barthel Index (ranging from 0 [total dependent] to 100 [independent]), Lawton and Brody Index (scores from 0 [dependent] to 8 [independent]), nutritional status (Mini Nutritional Assessment—Short Form), body composition (DXA), and quality of life (EQ-5D-5L) within 3 cohorts of older adults: hospital control cohort, hospital exercise cohort, and primary care cohort.

#### Secondary Outcomes

- In the hospital exercise cohort, we will assess changes in quantitative ultrasound and blood-based biomarkers, as well as clinical variables, following a 16-week multicomponent physical exercise program. Additionally, exploring associations between ultrasound and blood-based biomarkers and the remaining clinical variables both before and after the exercise program.
- In the hospital control cohort, we will examine changes in quantitative ultrasound and blood-based biomarkers, along with clinical variables, following a 16-week follow-up period without intervention. Furthermore, we will explore associations between quantitative ultrasound and blood-based biomarkers and the remaining clinical variables after the 16-week follow-up period.
- In the primary care cohort, we will assess changes in quantitative ultrasound and blood-based biomarkers, along with clinical variables, following a 1-year follow-up period without intervention. Additionally, we will explore associations between quantitative ultrasound and blood-based biomarkers and the remaining clinical variables after the 1-year follow-up.
- We will evaluate differences in the changes of all measurements among the 3 cohorts. A direct comparison will be made between the 2 hospital cohorts, while the primary care cohort will serve as a reference for understanding changes in nonhospital populations.

### Outcome Measurements

The list of clinical variables collected at baseline and follow-up assessments is included in Textbox 2.

**Clinical variables in baseline and follow-up examination.**

- Global Deterioration Scale of Reisberg for assessment of cognitive function.
- Charlson Comorbidity Index.
- Barthel Index of independence to perform basic activities of daily living.
- Lawton and Brody Index of independence to perform instrumental activities of daily living.
- Short Physical Performance Battery for Physical Function Assessment.
- SARC-F (Strength, Assistance with walking, Rising from a chair, Climbing stairs, and Falls) scale of sarcopenia screening.
- FRAIL (short 5-question assessment of Fatigue, Resistance, Aerobic capacity, Illnesses, and Loss of weight) scale of frailty evaluation.
- Frailty phenotype of frailty proposed and validated by Fried et al [4].
- Mini Nutritional Assessment—Short Form for nutritional screening.
- International Physical Activity Questionnaire for physical activity levels.
- EQ-5D-5L for health-related quality of life assessment.
- Gait Speed Test for Physical Function Assessment.
- Grip strength with Jamar dynamometer.
- Population characteristics: age, sex, and BMI.
- Body composition (dual-energy X-ray absorptiometry; total muscle mass, appendicular lean soft tissue mass [ALM], dual-energy X-ray absorptiometry [ALM/h^2^ or ALM divided by height squared], total fat, and fat percentage)

### Ultrasound Imaging and Raw Data Acquisition

The ultrasound equipment used in this study is the L7 HD3 portable linear scanner (Clarius Mobile Health Corp.). Specifically designed for point-of-care ultrasound examinations, the probe of this device boasts a frequency range of 4-13 MHz with a center frequency of 7 MHz. To facilitate seamless data transfer, anonymized digital ultrasound data are transmitted from the scanner through a customized Wi-Fi network to a smart device, enabling real-time B-mode navigation and data storage. The important feature of the scanner is an integrated image processing package designed to optimize B-mode musculoskeletal image quality. This technology enhances the clarity and quality of musculoskeletal imaging during examinations. Additionally, the scanner is equipped with a research package designed to capture raw beamformed backscattering ultrasound data after the beamformer. These raw data are presented in an in-phase/quadrature complex baseband representation, commonly referred to as IQ data. The IQ data serve as the foundation for the implementation of tailored quantitative ultrasound algorithms [71].

**Figure 3:**
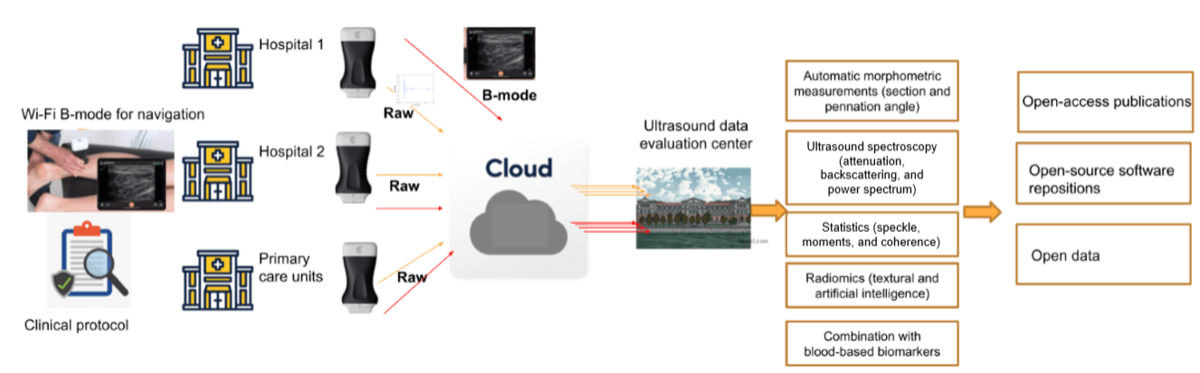
Ultrasound data flow and management from clinical acquisition centers to ultrasound data evaluation center.

All examinations will be conducted within a depth range of 0-60 mm, using 50% of the maximum acoustic output offered by the scanner. During measurements, efforts will be made to minimize skin-probe compression to maintain acceptable image quality. Following each examination, the acquired data will be uploaded to a HIPAA (Health Insurance Portability and Accountability Act)-compliant cloud service, offered by the scanner’s manufacturer, enabling the centralization of data (Figure 3).

### Ultrasound Examination Protocol

**Figure 4:**
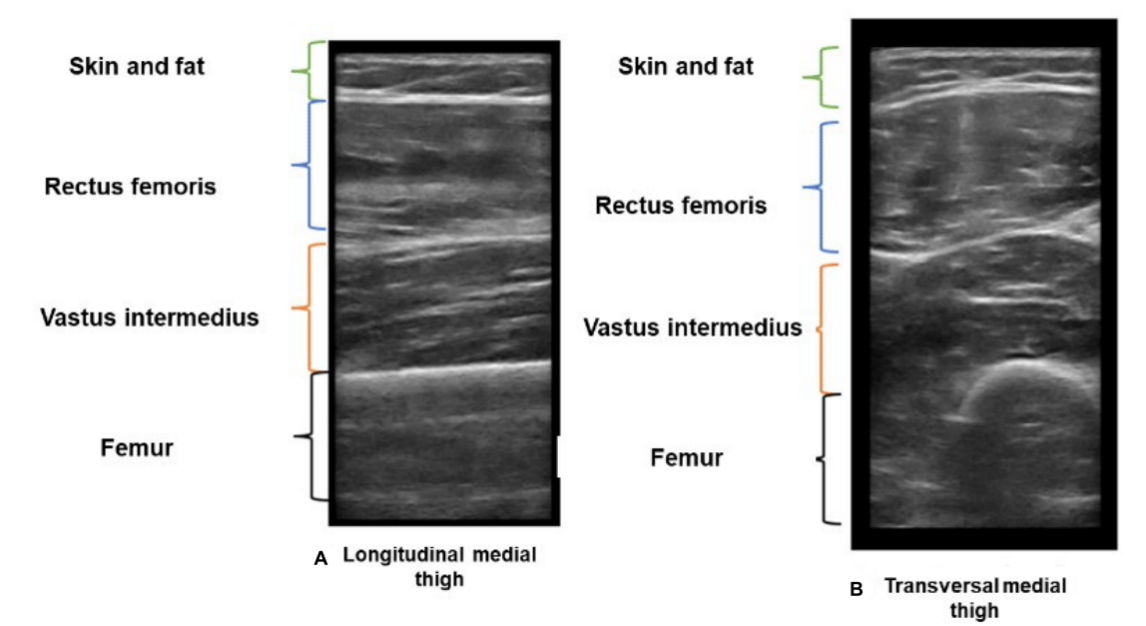
Echographic B-mode appearance of mid-thigh in the (A) transverse and (B) longitudinal views.

The midpoint of the thigh will be determined and marked as the half-distance between the superior border of the patella and the anterior-superior iliac crest. Using the femur as a guide in the transverse view, the midpoint of each thigh will be located, and the various components of the quadriceps muscles (rectus femoris, vastus intermedius, vastus medialis, and vastus lateralis) will be identified (Figure 4).

In each examination, a minimum of 12 coregistered B-scan and raw data frames will be obtained. This will include 3 transverse and 3 longitudinal views for both thighs. The longitudinal examination will be conducted in a plane where fasciculations are visible, ensuring comprehensive coverage and detailed assessment of the musculoskeletal structures.

### Evaluation of Quantitative Ultrasound Biomarkers

#### Morphometric Ultrasound Measures

**Figure 5:**
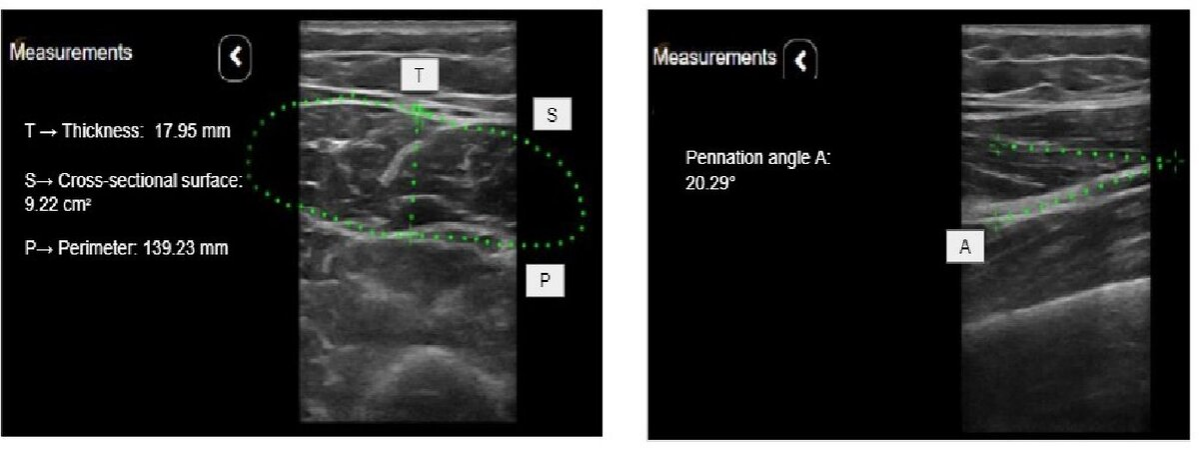
Morphometric ultrasound measurements. (A) Rectus femoris thickness, cross-sectional surface, and perimeter (transverse view). (B) Pennation angle (longitudinal view).

Throughout the examination, the sonographer will record morphometric ultrasound measurements using the online ultrasound scanner’s interface (Figure 5). This includes capturing the thickness of the rectus femoris in the transverse view at the midsection point, measuring the perimeter and cross-sectional area of the rectus femoris in the transverse view, and determining the pennation angle in the longitudinal view.

#### Raw Data Evaluation

Quantitative ultrasound biomarkers will be assessed offline using the acquired raw IQ data. Regions of interest encompassing the rectus femoris will be delineated in the raw data domain by automatically generating B-mode images from the raw data. This process will use the coregistered clinical B-mode images and sonographer annotations as a reference for the definition of the accurate region of interest. Table 1 presents a list of state-of-the-art quantitative ultrasound biomarkers, the hyperparameters of which will be systematically adjusted through experimental adaptation for musculoskeletal examinations. These adjustments will be made and subsequently evaluated using the raw data obtained from the ultrasound probes during the study.

**Table 1:** Quantitative ultrasound biomarkers based on raw data.

Biomarker	Description	Literature references
Automatic morphometric measurements	Automatic muscle morphometric analysis based on neural network segmentation models trained with respect to sonographer annotations of rectus femoris cross section, and 2D Fourier analysis of pennation angle.	[72,73]
Attenuation coefficient	Measurement of loss of signal intensity with depth.	[47,48,50]
Backscattering coefficient	Measurement of tissue reflectivity after attenuation compensation.	[74,75]
Power spectrum (Lizzi-Feleppa parameters)	Spectroscopy measurement of backscattered signal variation with frequency, including parametrizations such as spectral slope, spectral intercept, and midband fit	[49]
Speckle statistics	Fitting of raw envelope signal to speckle statistical distribution models, including Rayleigh, Nakagami, and homodyned K-distribution. Estimation of scatterer concentration, spacing, and coherence from fitted model parameters.	[49,76,77]
Statistical moments	Nonparametric statistical moments capturing scatterer distribution and concentration, such as entropy, kurtosis, skewness, variance, anisotropy, and signal-to-noise ratio.	[78,79]
Coherence and speed of sound	Generalized spectrum analysis and estimation of coherence, mean scatterer spacing, and speed-of-sound in muscle.	[80,81]
Textural radiomics	First- and second-order texture features extracted from both B-mode (eg, based on gray-scale co-occurrence matrices) and raw data (eg, based on wavelet and Laplacian transformations) and combined with machine learning models trained with respect to clinical outcomes.	[51,53,54]
Artificial intelligence radiomics	Radiofrequency data and B-mode features extracted automatically with end-to-end neural network models trained with respect to clinical outcomes	[52,82-84]

### Evaluation of Blood-Based Biomarkers

**Figure 6:**
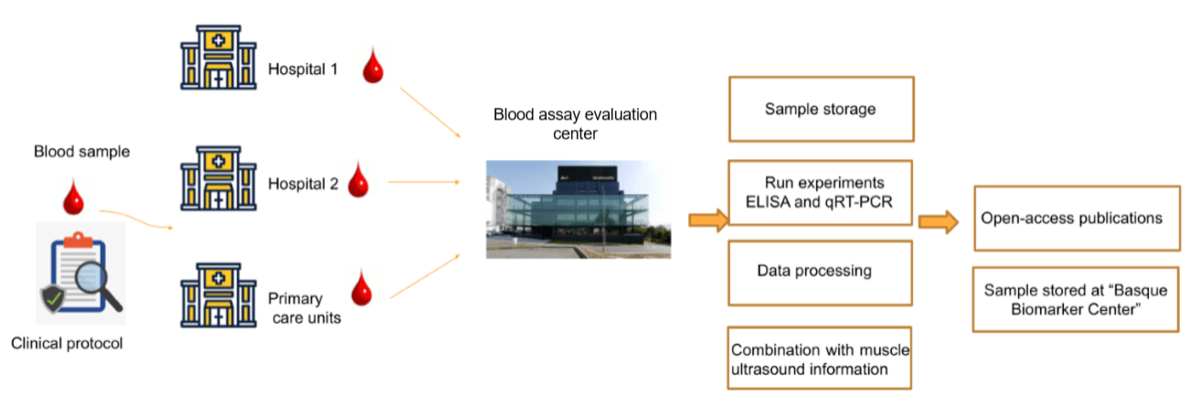
Biomarkers data flow and management from clinical centers to blood-essay evaluation center. ELISA: enzyme-linked immunosorbent assay; qRT-PCR: real-time quantitative reverse transcription polymerase chain reaction.

During both basal and follow-up acquisitions, venipuncture will be performed to collect one 10-mL serum blood tube and two 5-mL ethylenediaminetetraacetic acid (EDTA) blood tubes. The serum sample will undergo centrifugation and will be stored in 4 aliquots. One of the EDTA samples will be promptly frozen and stored at –80°C, while the other will be subjected to centrifugation for plasma and buffy coat extraction. These samples will be stored at –80°C at the clinical sites until transportation, which will be carried out with dry ice to the Blood-Assay Evaluation Center for subsequent processing and storage (Figure 6).

The expression of previously described biomarkers, including vitamin D, lutein zeaxanthin, troponin T, pro-brain natriuretic peptide, soluble receptor for advanced glycation end products (sRAGE) [85], and microRNAs, along with those associated with relevant pathways related to frailty such as inflammation (interleukin 6) and senescence (p16INK4A and p21CIP [56]), will be assessed in cells obtained from a blood sample. This evaluation will be conducted in a subsample of patients in baseline conditions and after intervention at both the transcriptional and protein levels, as outlined in Table 2. Erythrocytes will be lysed using Buffer EL (Qiagen), and total RNA from leukocytes will be isolated using the miRNeasy Mini Kit (Qiagen). Initially, RNA samples will undergo purification with the RNeasy Kit (Qiagen). Subsequently, the RNA will be retro-transcribed, and gene expressions will be quantified through quantitative polymerase chain reaction (PCR) using specific primers or probes and the ABI Prism SDS 7300 Real-Time PCR System (Applied Biosystems). Expression levels will be normalized to the expression of the enzyme glyceraldehyde-3-phosphate dehydrogenase (GAPDH) [86]. Furthermore, protein levels will be assessed in serum samples using Quantikine enzyme-linked immunosorbent assays and Luminex, as previously outlined [87].

**Table 2:** Blood-based biomarkers.

Biomarker	Method of measurement
Vitamin D	ELISA^a^
Lutein zeaxanthin	ELISA
Troponin T	ELISA
Pro-brain natriuretic peptide	ELISA
Soluble receptor for advanced glycation end products	ELISA
MicroRNA 125	qRT-PCR^b^
MicroRNA 194	qRT-PCR
MicroRNA 454	qRT-PCR
Interleukin-6	ELISA and Luminex
P16^INK4A^	qRT-PCR
P21^CIP^	qRT-PCR
New unpublished	qRT-PCR and ELISA

^a^ELISA: enzyme-linked immunosorbent assay.

^b^qRT-PCR: real-time quantitative reverse transcription polymerase chain reaction.

### Statistical Analysis

The study population will be characterized based on demographic and clinical attributes. Categorical data will be summarized with frequency and percentage representations. Continuous data will be described using mean, SD, median, and the 25th and 75th percentiles. Furthermore, the distribution of ultrasound parameters with respect to the primary endpoint will be depicted using graphical techniques. The correlation of quantitative biomarkers will be evaluated for each multifactorial clinical evaluation parameter using the Pearson correlation coefficient for continuous variables and the Spearman correlation coefficient for categorical variables [88]. Unpaired tests, such as the Student *t* test, will be used for comparing means of biomarkers among various acquired patient cohorts and for stratified patient subgroups based on the frailty scales outlined in Textbox 1. To manage the extensive array of ultrasound and blood biomarkers, a robust statistical analysis plan will be implemented. This plan will incorporate correction methods to address the risk of type I errors associated with multiple comparisons. Specifically, for the primary outcome concerning biomarker-clinical associations, we will use Lasso regression. This technique automates the selection of relevant variables, reducing the necessity for extensive corrections and improving interpretability. Paired statistics will be applied in the statistical analysis for longitudinal monitoring, encompassing both interventions and clinical follow-up. The monitoring period in hospital and primary care environments will differ to accommodate the distinct follow-up workflows in each setting. The duration of primary care follow-up aligns with established norms [89,90]. To account for differences in follow-up duration among cohorts over time, our data analysis will incorporate appropriate statistical methods, potentially enabling adjustments for these variations. For the secondary objective, the impact of physical intervention in 50 patients will be compared with 50 patients with no intervention. With 80% power, the planned population size will enable the detection of a biomarker with a medium effect size of E/S=0.284 at a 2-sided α level of .05.

In the context of multiparametric data science models, unsupervised learning techniques will be assessed to cluster patient populations based on biomarker expression. Moreover, supervised multi-omics models will be trained using a training set of clinical variables. Stratified cross-validation will be used to derive model performance statistics, ensuring separate patient data in the training and validation folds and maintaining a balanced distribution of health participants and those with frailty in both training and validation sets. Consecutively, sample imputation will be used to address missing data in the training set, while missing reference variables will be excluded during validation and testing. Sensitivity analyses will be conducted to evaluate the impact of the imputation method on the robustness of our results. Reserve data sets, comprising data from various sites and follow-up examinations, will be used for model testing.

Biomarker reproducibility will be evaluated using the interclass correlation coefficient and the Bland-Altmann method, taking into account repeated measurements within an examination. Subgroup analyses will be conducted to explore variations across patient subpopulations, and regression models will be used to examine the influence of covariates, thereby enhancing methodological precision.

### Ethical Considerations

This study protocol received approval from the Research Ethics Committee of Albacete (Spain) with reference CEIm-2021-51. The study will be conducted in adherence to the principles outlined in the Declaration of Helsinki [91]. Before participation, written informed consent will be obtained from all participants, and their data will be handled in accordance with HIPAA guidelines. Additionally, consent will be sought for the review of participants’ medical records and for the collection of blood samples to assess biomarkers. In the event of necessary modifications to this protocol during the clinical research, the changes will undergo review by the hospital ethics committee and will be implemented only after obtaining approval. The trial results will be communicated to participants via email by the investigators. Participants involved in this study will not receive any form of compensation for their participation.

The study information will be stored in Microsoft Excel 365 within an electronic database. This database will comprehensively capture individual data, including baseline characteristics, pre- and postassessment data, and any potential adverse events. Notably, no personally identifiable patient information, apart from the unique trial identification number, will be included in the metadata associated with the recorded ultrasound and biomarker data. Ultrasound and biomarker data will be uploaded to a HIPAA-compliant cloud service provided by the ultrasound system manufacturer, enabling centralized evaluation by team members. As outlined in the reference manuscript, upon completion of the study, digitized data sets encompassing ultrasound data, biomarkers, digitized blood-based biomarkers, and anonymized multifactorial clinical evaluations will be published in open-access repositories, such as Zenodo (CERN). Biological samples will be securely stored in the Basque Biomarker Center (Biobanco). Additionally, software models derived from ultrasound raw data will be made accessible through open-source software repositories (eg, GitLab).

## Results

The initial analysis results indicate a correlation between ultrasound morphometric parameters and clinical variables (Table 3). This table examines the correlations of ultrasound geometric parameters with clinical variables. The clinical data considered in this analysis are derived from a subset of the hospital cohort, comprising a total of 66 participants. Specifically, correlations between clinical variables and ultrasound parameters during the basal examination have been included in the table.

**Table 3:** Preliminary correlations at baseline visit for the hospital cohort (n=66).

Correlations	Thickness run average, *r* (*P* value)	Area run average, *r* (*P* value)	Angle run average, *r* (*P* value)
Age	–0.023 (.82)	0.002 (.98)	–0.072 (.47)
BMI	0.269 (.009)^a,b^	–0.016 (.88)	0.167 (.09)
Charlson	–0.180 (.09)	–0.213 (.04)^a,b^	–0.018 (.85)
Barthel Index	0.058 (.58)	0.176 (.09)	0.224 (.02)^a,b^
Lawton and Brody Index	–0.002 (.98)	–0.056 (.60)	0.220 (.02)^a,b^
Mini Nutritional Assessment—Short Form	0.183 (.08)	0.036 (.74)	0.210 (.03)^a,b^
SARC-F^c^	–0.098 (.35)	–0.236 (.02)^a,b^	–0.158 (.11)
Grip strength	0.240 (.02)^a,b^	0.372 (<.001)^a,b^	0.105 (.32)
Short Physical Performance Battery total	0.288 (.005)^a,b^	0.355 (.001)^a,b^	0.197 (.04)^a,b^
Gait speed	0.168 (.11)	0.327 (.002)^a,b^	0.241 (.01)^a,b^
Frailty phenotype (Fried et al [4])	–0.101 (.34)	–0.211 (.04)^a,b^	–0.044 (.68)
FRAIL^d^	–0.125 (.24)	–0.284 (.006)^a,b^	–0.034 (.73)
DXA^e^ total muscle mass	0.473 (<.001)^b,f^	0.498 (<.001)^b,f^	0.143 (.28)
DXA ALM^g^	0.440 (<.001)^b,f^	0.539 (<.001)^b,f^	0.100 (.45)
DXA ALM/h^2^	0.529 (<.001)^b,f^	0.493 (<.001)^b,f^	0.115 (.38)
DXA total fat	0.271 (.04)^a,b^	0.29 (.83)	0.130 (.32)
DXA fat percentage	0.132 (.31)	–0.239 (.07)	0.064 (.63)

^a^Significant weak correlations (*r*<0.4).

^b^Significant correlations (*P*<.05).

^c^SARC-F: Strength, Assistance with walking, Rising from a chair, Climbing stairs, and Falls.

^d^FRAIL: short 5-question assessment of Fatigue, Resistance, Aerobic capacity, Illnesses, and Loss of weight.

^e^DXA: dual-energy X-ray absorptiometry.

^f^Significant moderate and strong correlations (*r*>0.4).

^g^ALM: appendicular lean soft tissue mass.

The analysis revealed significant moderate correlations between muscle cross-section and thickness and DXA parameters (Table 3). Notably, the highest correlation (*r*=0.539, *P*<.001) was observed between DXA appendicular mass and muscle cross section. Additionally, significant weak correlations were identified between functional variables (such as gait speed, grip strength, and SPPB) and ultrasound morphometric parameters (including muscle section, thickness, and pennation angle; indicated in Table 3 in rows 7, 8, and 9, respectively). The frailty scale (FRAIL, Fried et al [4]) and the sarcopenia scale (SARC-F) exhibited weak but significant associations with muscle cross section as illustrated in row 2 of Table 3. Additionally, significant weak correlations were identified between pennation angle and measures of independence (Barthel and Lawton Index) as well as nutritional assessment (Mini Nutritional Assessment—Short Form) as detailed in Table 3. Notably, age did not demonstrate a significant association with ultrasound morphometric variables (*r*=–0.023, *P*=.82; *r*=0.002, *P*=.98; and *r*=–0.072, *P*=.47, respectively). However, a weak correlation was observed between BMI and muscle thickness.

## Discussion

### Principal Findings

The anticipated outcomes of this study are the identification of associations and the development of combination models between quantitative ultrasound and blood-based assays with multifactorial clinical assessments, specifically focusing on muscle quality and frailty. The use of noninvasive portable technologies facilitates the implementation of the clinical protocol in both hospital and primary care environments [92].

The primary findings concerning the correlations of ultrasound geometric variables with clinical and functional variables align with existing clinical literature [17,35,36,41]. These preliminary results are encouraging, demonstrating robust correlations between echo geometric parameters, measured in a standardized manner, and various clinical parameters. Notably, moderate correlations are observed between ultrasound geometrical parameters and muscle mass (DXA), while functional parameters and global scales of sarcopenia and frailty exhibit weak yet significant associations. These results serve as a foundational baseline for the development of advanced models based on raw data (Table 1) and blood biomarkers. It is anticipated that these advanced models will exhibit superior correlations with basal characteristics and enhanced sensitivity to changes in muscle quality.

### Comparison With Prior Work

Recent literature indicates that impaired mobility associated with aging is not solely attributed to changes in skeletal muscle mass; other factors contributing to muscle quality also play a crucial role. Notably, alterations in muscle tissue composition, characterized by elevated levels of intramuscular adipose tissue and intramyocellular lipids, have been identified as factors that negatively impact muscle functional capacity [17,65,93]. Our hypothesis suggests that quantitative ultrasound biomarkers derived from raw data may exhibit superior discriminative performance, greater reproducibility, and enhanced ease of use compared with the current state-of-the-art assessments based on B-mode images. B-mode morphological ultrasound parameters are primarily linked to muscle mass and demonstrate limited sensitivity in the diagnosis of sarcopenia. Quantitative ultrasound biomarkers derived from raw data have consistently demonstrated their ability to capture both tissue composition and microstructural properties. Furthermore, they have exhibited a greater capacity to encode richer information content compared with ultrasound B-mode images in artificial intelligence models, as highlighted in prior research [52,54,83,84]. Ultrasound spectroscopy parameters and tissue acoustic properties, such as speed of sound and attenuation, have been associated with tissue composition [47] and viscoelastic changes in muscle [49] among older individuals with sarcopenia. Specifically, speed of sound has demonstrated correlations with MRI adiposity estimates in the calf muscles [94], CT assessment of the psoas muscle [95], and short-term changes in muscle due to immobilization [96]. The ultrasound statistical analysis of the envelope signal in soft tissues has been correlated with the concentration, spacing, and directionality of microstructural scatterers [78]. Additionally, texture features derived from radiomics analysis capture musculoskeletal composition and microstructure. This approach has been successfully applied to differentiate various musculoskeletal conditions, including muscle spasticity, dynapenia, myositis, fibromyalgia, Duchenne muscular dystrophy, and exercise-induced muscle damage [55,97]. Being acquired at the early stages of ultrasound image formation, ultrasound raw data may play a crucial role in minimizing equipment- and operator-dependent bias. Additionally, they have the potential to provide data-based guidance for examiners with limited sonographic acquisition expertise.

Molecular biomarkers obtained from blood assays are linked to functional changes between healthy individuals and those with frailty. However, the absence of single established predictor biomarkers is primarily attributed to several factors. These include the heterogeneity and limitations of the scales or indices used to detect sarcopenia and frailty; variations in age, sex, and characteristics across different populations; small sample sizes; limited longitudinal clinical studies; a lack of characterization on interventions to assess their potential reversibility; and differences in techniques and cut-offs used for biomarker measurement. In this study, we incorporate a panel of biomarkers rather than assessing individual molecules. This approach is aimed at providing a more comprehensive reflection of the accumulation of damage associated with age-related syndromes [98]. Additionally, the potential of these biomarkers to assess reversibility, a critical characteristic of frailty, will be evaluated upon completion of an intervention based on an exercise program, followed by a 16-week follow-up.

Sarcopenia and frailty, although related, are distinct aging phenotypes. Notably, the prevalence of sarcopenia is higher among older individuals with frailty than the prevalence of frailty among those with sarcopenia [28-32]. As part of our exploratory outcomes, we will consider the coexistence of sarcopenia and frailty, redefining the frailty prognosis based on the presence or absence of sarcopenia.

To the best of our knowledge, this cohort study stands out as one of the few that integrates raw data ultrasound measurements with blood samples to extract noninvasive biomarkers of frailty and sarcopenia in older adults. Notably, this study represents the first of its kind to establish a connection between quantitative ultrasound and blood biomarkers with the cross-sectional evaluation of frailty and sarcopenia in both hospital and primary care environments. The utilization of point-of-care ultrasound devices presents an opportunity to introduce ultrasound quantitative technology across various clinical settings. Notably, this study marks the pioneering effort to combine quantitative ultrasound with blood-based biomarkers to evaluate musculoskeletal changes following multicomponent physical exercise programs.

### Limitations

The study does have certain limitations, notably the absence of access to a gold standard for muscle quality assessment. Established radiological techniques such as CT and MRI serve as references for muscle composition and microstructure but come with patient complications and are not widely accessible in the clinical environments under investigation. Instead, we rely on a multifactorial clinical assessment, patient stratification, and carefully controlled interventions to evaluate changes in muscle quality. The exploration of ultrasound technology is limited to backscattering ultrasound data derived from beamformed raw data. Other ultrasound quantitative technologies, such as shear wave elastography [43] and blood flow measurements based on Doppler sequences [99], are excluded from the scope because of their lack of routine availability in point-of-care ultrasound devices and the added complexity they introduce to the protocol execution. The disparate monitoring periods in hospital (16 weeks) and primary care environments (1 year) are designed to accommodate the distinct follow-up workflows in both settings. We also acknowledge that the randomization of populations in the secondary goal is conducted on an institutional basis, which could introduce statistical bias if the populations in the 2 institutions differ. To address this potential bias, we will perform a *post hoc* analysis to examine the characteristics of the populations in the various recruitment centers. The dimensioning of the primary care follow-up aligns with periods established in previous studies for the general population [89,90].

### Conclusions

In summary, the outlined study protocol aims to integrate portable technologies, leveraging quantitative muscle ultrasound and blood-based biomarkers, for an objective cross-sectional assessment of muscle quality in both hospital and primary care environments. The study endeavors to yield data that will facilitate the exploration of associations between biomarker combinations and the cross-sectional clinical evaluation of frailty and sarcopenia, alongside an examination of musculoskeletal changes following multicomponent physical exercise programs.

## Appendix

Multimedia Appendix 1 SPIRIT (Standard Protocol Items of the Recommendations for Interventional Trials) checklist.

## Abbreviations

CT: computed tomography
DOCS: Definition and Outcomes Consortium
DXA: dual-energy X-ray absorptiometry
EDTA: ethylenediaminetetraacetic acid
EWGSOP-2: European Working Group on Sarcopenia in Older People 2
FRAIL: short 5-question assessment of Fatigue, Resistance, Aerobic capacity, Illnesses, and Loss of weight
GAPDH: glyceraldehyde-3-phosphate dehydrogenase
HIPAA: Health Insurance Portability and Accountability Act
MRI: magnetic resonance imaging
NIHF: National Institute of Health Foundation
PCR: polymerase chain reaction
SARC-F: Strength, Assistance with walking, Rising from a chair, Climbing stairs, and Falls
SPIRIT: Standard Protocol Items of the Recommendations for Interventional Trials
SPPB: Short Physical Performance Battery
sRAGE: soluble receptor for advanced glycation end products (sRAGE)

## Availability of Data and Materials

The outcomes of this study will be disseminated through publication in an academic journal. Additionally, we plan to organize at least two interdisciplinary workshops involving geriatrics and imaging specialists. To promote transparency and encourage further research, digitalized data sets, comprising ultrasound data and biomarkers, digitized blood-based biomarkers, and multifactorial clinical evaluations, will be published in open-access repositories such as Zenodo. Biological samples will be securely stored at the Basque Biomarker Center (Biobanco). Furthermore, software models derived from ultrasound raw data will be made accessible through open-source software repositories such as GitLab.
