# Supplementary material for: Development of Continuous Assessment of Muscle Quality and Frailty in Older Patients Using Multiparametric Combinations of Ultrasound and Blood Biomarkers: Protocol for the ECOFRAIL Study
Source: JMIR Res Protoc. 2024 Feb 23;13:e50325. doi: 10.2196/50325 (PMC10924264; doi:10.2196/50325)
Supplement: Multimedia Appendix 1 [file resprot_v13i1e50325_app1.pdf]

SPIRIT Checklist

		Reporting Item	Page and Line Number	Reason if not applicable
**Administrative information**				
Title	#1	Descriptive title identifying the study design, population, interventions, and, if applicable, trial acronym	P1, line1-4	
Trial registration	#2a	Trial identifier and registry name. If not yet registered, name of intended registry	P2, line 63-65	
Trial registration: data set	#2b	All items from the World Health Organization Trial Registration Data Set	P2, line 63-65	
Protocol version	#3	Date and version identifier	P2, line 63-65	
Funding	#4	Sources and types of financial, material, and other support	P19, line 590-598	
Roles and responsibilities: contributorship	#5a	Names, affiliations, and roles of protocol contributors	P1, line6-30/P19, line 600-609	

Roles and responsibilities: sponsor contact information	#5b	Name and contact information for the trial sponsor	N/A	The University of Deusto is ultimately responsible for the research. The contact data (Xabier Rio de Frutos) reflected in the Clinical Trials represents the research department, also corresponding to one of the clinical researchers participating in the project on behalf of the University of Deusto. The project PI (lead author Sergio Sanabria) and deputy coordinator (Diego Lopez-de-Ipiña) are included in the protocol (PhD). The clinical principal investigators (PhD, MD) according to IRB submissions for each local clinical site are included in "Locations". The clinical Principal Investigator (PhD, MD) corresponds to the coordinating IRB submission (Albacete).
Roles and responsibilities: sponsor and funder	#5c	Role of study sponsor and funders, if any, in study design; collection, management, analysis, and interpretation of data; writing of the report; and the decision to submit the report for publication, including whether they will have ultimate authority over any of these activities	N/A	This is an investigator-initiated study, in which the University of Deusto is the sponsor. The funding agency is the Department of Health of the Basque Government within the Call for Grants for Research and Development Projects in Health (2021).
Roles and responsibilities: committees	#5d	Composition, roles, and responsibilities of the coordinating centre, steering committee, endpoint adjudication committee, data management team, and other individuals or groups overseeing the trial, if	P19, line 600-609.	Project Coordination: University of Deusto, Sergio Sanabria, PhD (Principal Investigator), Diego-Lopez-de Ipiña (Deputy). Monthly meetings with PIs of each clinical site to oversee conduct and progress.<br>Trial Steering Committee: Project progress is monitored by sponsor (Gobierno Vasco), annual reporting. Ethics monitoring is performed by CEIM (Comité Etico de Investigación con Medicamentos). Principal Clinical Investigator coordinating CEIM submission and patient recruitment/consent: Pedro Abizanda Soler - PhD, MD - Complejo Universitario de Albacete. Clinical Investigators local sites CEIM submission: Leocadio Rodriguez Mañas - University Hospital of Getafe, PhD, MD. Itziar Vergara - PhD, MD; Ander Matheu - PhD - Biodonostia Health Research

		applicable (see Item 21a for data monitoring committee)		Institution. Each clinical investigator oversees conduct and progress of clinical teams at each respective site.<br>SPIG: Not applicable
**Introduction**				
Background and rationale	#6a	Description of research question and justification for undertaking the trial, including summary of relevant studies (published and unpublished) examining benefits and harms for each intervention	P3-5, line 68-157	
Background and rationale: choice of comparators	#6b	Explanation for choice of comparators	P5,line146-153	
Objectives	#7	Specific objectives or hypotheses	P5,line154-157	
Trial design	#8	Description of trial design including type of trial (eg, parallel group, crossover, factorial, single group), allocation ratio, and framework (eg, superiority, equivalence, non-inferiority, exploratory)	P5-6, line160-168	
**Methods: Participants, interventions, and outcomes**				

Study setting	#9	Description of study settings (eg, community clinic, academic hospital) and list of countries where data will be collected. Reference to where list of study sites can be obtained	P5-6, line160-193	
Eligibility criteria	#10	Inclusion and exclusion criteria for participants. If applicable, eligibility criteria for study centres and individuals who will perform the interventions (eg, surgeons, psychotherapists)	P6,line185-213	
Interventions: description	#11a	Interventions for each group with sufficient detail to allow replication, including how and when they will be administered	P7, line229-245	
Interventions: modifications	#11b	Criteria for discontinuing or modifying allocated interventions for a given trial participant (eg, drug dose change in response to harms, participant request, or improving / worsening disease)	P7, line 221-223	

Interventions: adherance	#11 c	Strategies to improve adherence to intervention protocols, and any procedures for monitoring adherence (eg, drug tablet return; laboratory tests)	P8, line 246-260	Monitoring by physicians responsible for each patient, geriatrician monitoring progress
Interventions: concomitant care	#11 d	Relevant concomitant care and interventions that are permitted or prohibited during the trial	P7, line 225-227	All participants will receive care or interventions as usual
Outcomes	#12	Primary, secondary, and other outcomes, including the specific measurement variable (eg, systolic blood pressure), analysis metric (eg, change from baseline, final value, time to event), method of aggregation (eg, median, proportion), and time point for each outcome. Explanation of the clinical relevance of chosen efficacy and harm outcomes is strongly recommended	P9-13, line296-400	
Participant timeline	#13	Time schedule of enrolment, interventions (including any run-ins and washouts), assessments, and visits for	P5-6, line 166-183	Figure 1

		participants. A schematic diagram is highly recommended (see Figure)		
Sample size	#14	Estimated number of participants needed to achieve study objectives and how it was determined, including clinical and statistical assumptions supporting any sample size calculations	P8-9,line280-294	
Recruitment	#15	Strategies for achieving adequate participant enrolment to reach target sample size	P6,line188-193	
**Methods: Assignment of interventions (for controlled trials)**				
Allocation: sequence generation	#16a	Method of generating the allocation sequence (eg, computer-generated random numbers), and list of any factors for stratification. To reduce predictability of a random sequence, details of any planned restriction (eg, blocking) should be provided in a separate document that is unavailable to those who	p7, line 225-227.	The patients will be randomly recruited in each centre sequentially in each centre according to the scheduled patient lists. The way to code the participants is two digits indicating the centre number (XX) followed by two digits indicating the patient number (YY), all consecutively according to recruitment. The code would be XX/YYY. We do not include stratification in the recruitment, apart from the different center allocation (primary care, hospital units). Post hoc, we will stratify patients in robust/frail groups according to clinical characteristics and frailty scale of Table 1. This information will be evaluated a posteriori, so it will be unavailable for enrolment or assignment of interventions.

		enrol participants or assign interventions		
Allocation concealment mechanism	#16b	Mechanism of implementing the allocation sequence (eg, central telephone; sequentially numbered, opaque, sealed envelopes), describing any steps to conceal the sequence until interventions are assigned	p7, line 215-227	The allocation sequence in this multi-centric study is designed on an institution-based basis. Patients recruited at the Department of Geriatrics of the Complejo Hospitalario Universitario de Albacete will be allocated by default to the supervised exercise program. Patients recruited at the Geriatrics Department of the University Hospital of Getafe will not be allocated to interventions. Similar socio-demographic characteristics are expected for both patient populations. In each institution, patients will be recruited from the falls unit, outpatient clinics and day hospital. Within each acquisition site, patient selection will be sequentially performed based on the clinical agenda up to the completion of the recruitment goals.
Allocation: implementation	#16c	Who will generate the allocation sequence, who will enrol participants, and who will assign participants to interventions	P7, line 225-228	
Blinding (masking)	#17a	Who will be blinded after assignment to interventions (eg, trial participants, care providers, outcome assessors, data analysts), and how	P8,line270-278	
Blinding (masking): emergency unblinding	#17b	If blinded, circumstances under which unblinding is permissible, and procedure for revealing a participant's allocated intervention during the trial	N/A	The design is open label so unblinding will not occur.

Methods: Data collection, management, and analysis				
Data collection plan	#18 a	Plans for assessment and collection of outcome, baseline, and other trial data, including any related processes to promote data quality (eg, duplicate measurements, training of assessors) and a description of study instruments (eg, questionnaires, laboratory tests) along with their reliability and validity, if known. Reference to where data collection forms can be found, if not in the protocol	P9-12, line280-353	
Data collection plan: retention	#18 b	Plans to promote participant retention and complete follow-up, including list of any outcome data to be collected for participants who discontinue or deviate from intervention protocols	P7-8, line 229-259	patients will be recommended for additional measures aimed at preventing weakness and risk of falls, and discontinued when medical care interferes with correct conduct of the study
Data management	#19	Plans for data entry, coding, security, and storage, including any related processes to promote data quality (eg, double data entry;	P8 line 270-278. P19 line 573-583	Biological samples will be stored in the Basque Biomarker Center (Biobanco). The serum sample will be centrifuged and stored in four aliquots. One of the EDTA samples will be immediately frozen and stored at -80ºC, and the other one will be centrifuged for plasma and buffy coat extraction. Samples will be stored at -80ºC at the clinical sites, before transportation with dry ice to the Blood-Essay

		range checks for data values). Reference to where details of data management procedures can be found, if not in the protocol		Evaluation Center for subsequent processing and storage. For the management, monitoring and security in the quality of the ultrasound data, the commercial platform clarius (brand of ultrasound scanners used in the project) will be used.
Statistics: outcomes	#20a	Statistical methods for analysing primary and secondary outcomes. Reference to where other details of the statistical analysis plan can be found, if not in the protocol	P14 line 401-423	
Statistics: additional analyses	#20b	Methods for any additional analyses (eg, subgroup and adjusted analyses)	N/A	The data in this study does not require subgroup analysis
Statistics: analysis population and missing data	#20c	Definition of analysis population relating to protocol non-adherence (eg, as randomised analysis), and any statistical methods to handle missing data (eg, multiple imputation)	P9, line 288-294. P14 line 401-423	10% drop-outs are foreseen and accounted for in the sample size calculation. data from patients with missing follow-up data will be included for randomised analysis of the primary outcomes (page 9).<br><br>Random sample imputation will be used for missing data in training, and missing reference variables will be excluded from validation and testing. (see Statistical analysis page 13-14).
**Methods: Monitoring**				
Data monitoring:	#21a	Composition of data monitoring committee (DMC); summary of its role and	P8, line 261-278.	Safety aspects will be monitoring by the principal investigator of each clinical site (see safety monitoring page 7). Data allocation in training and validation sets will be blinded to data evaluation centers (see randomization and blinding Page 6).

formal committee		reporting structure; statement of whether it is independent from the sponsor and competing interests; and reference to where further details about its charter can be found, if not in the protocol. Alternatively, an explanation of why a DMC is not needed		
Data monitoring: interim analysis	#21b	Description of any interim analyses and stopping guidelines, including who will have access to these interim results and make the final decision to terminate the trial	P8, line 272-278.	
Harms	#22	Plans for collecting, assessing, reporting, and managing solicited and spontaneously reported adverse events and other unintended effects of trial interventions or trial conduct	p8, line 261-268.	Participation in an exercise program is associated with a low risk (<1%) of adverse events (Singh et al., 2020), and most are usually low-grade responses to exercise, e.g. muscle soreness (Valenzuela et al., 2020).However, if adverse events or serious adverse events occur, they will be reported to the CEIMs.
Auditing	#23	Frequency and procedures for auditing trial conduct, if any, and whether the process will be independent from investigators and the sponsor	P20, line 596-598.	As part of grant specifications, yearly reports are submitted to the funding institution. As part of IRB specifications, auditing may be performed from the ethics committee during the study.

Ethics and dissemination				
Research ethics approval	#24	Plans for seeking research ethics committee / institutional review board (REC / IRB) approval	p19, line 555-562	
Protocol amendments	#25	Plans for communicating important protocol modifications (eg, changes to eligibility criteria, outcomes, analyses) to relevant parties (eg, investigators, REC / IRBs, trial participants, trial registries, journals, regulators)	P18, line 560-562.	If it is necessary to modify this protocol during clinical research, it will be reviewed by the hospital ethics committee and implemented after approval. The investigators will communicate the trial results to participants via email.
Consent or assent	#26a	Who will obtain informed consent or assent from potential trial participants or authorised surrogates, and how (see Item 32)	P6, line 188-193	Recruitment is carried out by the study coordinator staff in each site, and ultimately supervised by the clinical principal investigator at each site. The informed consent will be signed by the participant at the beginning of the first visit. The professional in charge of the assessment will explain the project and the participant will sign the information sheet and the informed consent to participate in the project. The principal investigator at each site will collect and monitor the informed consent documentation. Appendix 1.
Consent or assent: ancillary studies	#26b	Additional consent provisions for collection and use of participant data and biological specimens in ancillary studies, if applicable	P18, line 557-560.	Applicable in appendix 1.<br>"We will request consent for review of participants' medical records, and for the collection of blood samples to assess biomarkers

Confidentiality	#27	How personal information about potential and enrolled participants will be collected, shared, and maintained in order to protect confidentiality before, during, and after the trial	P19, line 573-585..	“No identifiable patient information apart from the individual trial identification number will be included in the metadata of the recorded ultrasound and biomarker data. Ultrasound and biomarker data will be uploaded to a HIPAA -compliant cloud service provided by the ultrasound system manufacturer for centralised evaluation by the team members of the data evaluation centers.<br><br>At indicated in (reference manuscript), at the end of the study we will publish digitalized datasets, including ultrasound data and biomarkers, digitised blood-based biomarkers and anonymized multi-factorial clinical evaluation in open-access repositories (e.g., Zenodo). Biological samples will be stored in the Basque Biomarker Center (Biobanco). Software models derived from the ultrasound raw data will be made available in open-source software repositories (e.g., GitLab).”
Declaration of interests	#28	Financial and other competing interests for principal investigators for the overall trial and each study site	P20,line 587	
Data access	#29	Statement of who will have access to the final trial dataset, and disclosure of contractual agreements that limit such access for investigators	P19,line573-585.	
Ancillary and post trial care	#30	Provisions, if any, for ancillary and post-trial care, and for compensation to those who	N/A	there will be no compensation

		suffer harm from trial participation		
Dissemination policy: trial results	#31a	Plans for investigators and sponsor to communicate trial results to participants, healthcare professionals, the public, and other relevant groups (eg, via publication, reporting in results databases, or other data sharing arrangements), including any publication restrictions		
Dissemination policy: authorship	#31b	Authorship eligibility guidelines and any intended use of professional writers	P20, line 600-609.	Authorship will be decided before publications depending on the researchers´ contributions to the specific manuscripts.
Dissemination policy: reproducible research	#31c	Plans, if any, for granting public access to the full protocol, participant-level dataset, and statistical code	P18,line 564.571	Free access to the journal will be paid for and all data codes and databases will be made available. Software models derived from the ultrasound raw data will be made available in open-source software repositories (e.g., GitLab).
**Appendices**				
Informed consent materials	#32	Model consent form and other related documentation given to participants and authorised surrogates	Appendix 1	Appendix 1. Appendix 1:<br><br>Translated to English from original protocol.

INFORMED CONSENT

Title of the study: "Development of continuous assessment of muscle fragility and quality in older subjects using multiparametric omics based on ultrasound and blood analysis". ECOFRAIL study.

TO BE FILLED IN ONLY BY THE PATIENT (in his/her own handwriting):

I (name and surname) ____

I have read and understood the information sheet given to me.
I have been able to ask questions about the study and they have been answered.
I have received sufficient information about the study.
Destination of the leftover sample after use in this research project

- ☐ **Destruction**
- ☐ **Donate the surplus sample to the Basque Biobank for Research of the Basque Foundation for Health Innovation and Research (BIOEF) to be kept and used for future research.**
- ☐ **Coded (to know the results of the research carried out).**
- ☐ **Anonymised (nobody can ever recover the link between your samples and you).**

I have spoken to: ____
(Name of Researcher or person designated to provide the information)

I understand that my participation is voluntary.
I understand that I can withdraw from the study:
- Whenever I want.
- Without having to explain myself.
- Without affecting my medical care.

I freely agree to participate in the study.

Date: Signature of Participant
(To be completed by the patient)

<table>
<tr><td></td><td></td><td></td><td></td><td>Date: Signature of the person explaining the informed consen

Patient Information Sheet Version 3, May 31, 2022. 2</td></tr>
<tr><td>Biological specimens</td><td>#33</td><td>Plans for collection, laboratory evaluation, and storage of biological specimens for genetic or molecular analysis in the current trial and for future use in ancillary studies, if applicable</td><td>P11, line 334-340.

Appendix 2</td><td>Appendix 2:

Ultrasound data flow and management from clinical acquisition centres to ultrasound data evaluation centre.

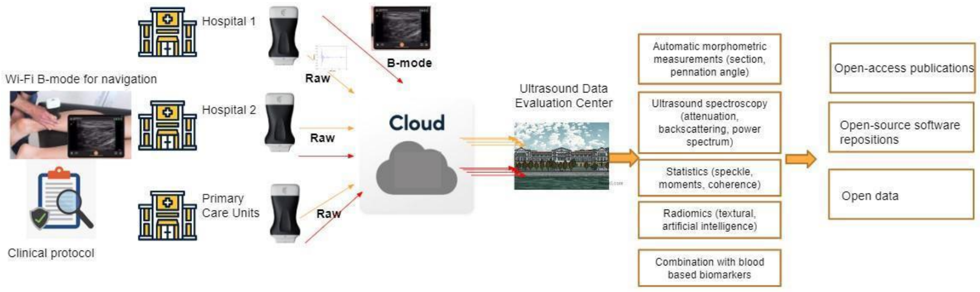

Biomarkers data flow and management from clinical centres to blood-essay evaluation centre.</td></tr>
</table>

				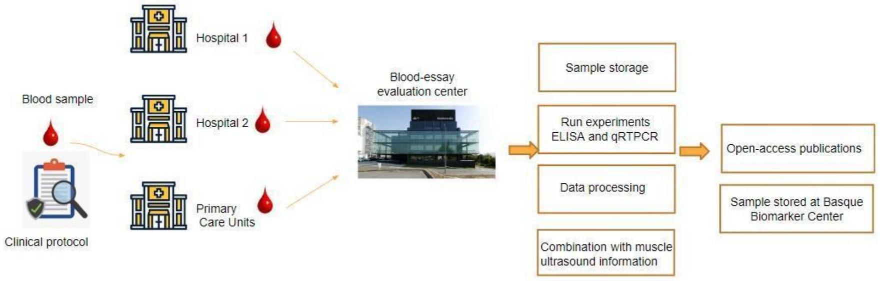

It is strongly recommended that this checklist be read in conjunction with the SPIRIT 2013 Explanation & Elaboration for important clarification on the items. Amendments to the protocol should be tracked and dated. The SPIRIT checklist is copyrighted by the SPIRIT Group under the Creative Commons "Attribution-NonCommercial-NoDerivs 3.0 Unported" license. This checklist can be completed online using https://www.goodreports.org/, a tool made by the EQUATOR Network in collaboration with Penelope.ai
